# Surgical management practice of pelvic organ prolapse among Ethiopian gynecologists, 2021: a descriptive study

**DOI:** 10.1186/s12905-023-02818-x

**Published:** 2023-12-08

**Authors:** Zelalem Ayichew Workineh, Birhanu Abera Ayana, Kiros Terefe Gashaye, Solomon Berhe Wubneh, Belayneh Ayanaw Kassie

**Affiliations:** 1 Department of Gynecology and Obstetrics, School of Medicine, College of Medicine and Health Sciences, University of Gondar, Gondar, Ethiopia; 2 Department of Women’s and Family Health, School of Midwifery, College of Medicine and Health Sciences, University of Gondar, Gondar, Ethiopia

**Keywords:** Ethiopian gynecologists, Pelvic organ prolapse, Surgical management practice

## Abstract

**Introduction:**

Pelvic Organ Prolapse is the descent of one or more of the anterior vaginal wall, posterior vaginal wall, the uterus, or the apex of the vagina. Surgical intervention addresses both anatomical defect and associated symptoms. The landscape of prolapse surgery has been evolving constantly over years. Emerging evidences either support or challenge existing surgical treatment options, making urogynecology a dynamic field. In Ethiopia, the surgical management of pelvic organ prolapse has transitioned from abdominal to vaginal hysterectomy, supplemented later by McCall’s culdoplasty. Disparities exist in the national uniformity of surgical approaches, linked to the establishment of Urogynecology centers in certain institutions.

**Objectives:**

This study was done to assess the surgical management practice of Ethiopian gynecologists on pelvic organ prolapse.

**Methods:**

A cross-sectional study encompassing all Gynecologists in Ethiopia took place between January to June 2021. Information was gathered through online Google forms crafted in English. Subsequently, the collected data underwent verification, coding, and entry into Epi info 7 before being exported to SPSS version 22 software for descriptive statistical analysis.

**Results:**

We reached 280 gynecologists out of the 450 practicing in the Ethiopia making 62% response rate. Anterior colporrhaphy (98.6%), vaginal hysterectomy with McCall’s cul-do-plasty (51.8%), and Posterior colporrhaphy (97.5%) were the most commonly performed surgical procedures for anterior vaginal wall prolapse, apical prolapse (uterine/cervical), and posterior vaginal wall prolapse respectively. Only 3.2% and 0.7% of the gynecologists conducted abdominal and vaginal paravaginal repair for anterior vaginal wall prolapse. Sacrospinous ligament fixation and sacrocolpopexy for apical prolapse were carried out by 32.9% and 9.3% of the gynecologists respectively. Site-specific posterior repair for posterior vaginal wall prolapse was performed only by 23.9% of the gynecologists. The main reasons mentioned not to perform paravaginal repair, sacrocolpopexy, sacrospinous ligament fixation, and site-specific posterior repair were lack of skill and lack of appropriate materials.

**Conclusion and recommendation:**

Most gynecologists in Ethiopian continue to perform vaginal hysterectomy and colporrhaphy procedures for treatment of pelvic organ prolapse due primarily to lack of skill and appropriate materials to perform the alternative procedures. Implementing short term training on alternative surgical treatment options of pelvic organ prolapse with provision of suitable materials and increasing the number of urogynecologists in the country in the long run holds the potential to enhance the standard of care of women with the condition.

**Supplementary Information:**

The online version contains supplementary material available at 10.1186/s12905-023-02818-x.

## Introduction

Pelvic Organ Prolapse (POP) is the descent of one or more of the anterior vaginal wall, posterior vaginal wall, the uterus (cervix) or the apex of the vagina (vaginal vault or cuff scar after hysterectomy) [1]. In Ethiopia, the problem is prevalent in higher magnitude with an estimated national prevalence of 23.52% [2, 3]. Surgery is one of the treatment options which will improve both anatomic problems and the symptoms related to them. Prolapse surgery has been changing constantly over the last few years as there is growing evidence in support or against the existing management options as well as emerging trends [4]. Adequate support of the apical defect is believed to be an important component of a durable repair for women with advanced prolapse [5]. Sacrospinous ligament fixation, uterosacral ligament suspension, McCall’s Cul-do-plasty, transvaginal mesh, and sacral colpopexy are options of management for an apical defect [6]. For anterior vaginal wall prolapse, paravaginal repair (transvaginal or retro pubic), anterior colporrhaphy (vaginal or transabdominal), concomitant anterior and paravaginal repair when both central and lateral defects are encountered are the options of surgical treatment [7]. The options for posterior wall defect repair include midline plication (posterior colporrhaphy), site-specific technique, graft/mesh augmentation of midline or site-specific repairs, transanal repair, ventral rectopexy, and sacral colpopexy in which mesh is extended to the distal portion of the posterior vaginal wall and/or perineum [8, 9]. Although the use of meshes in anterior an posterior compartment defect repair results in good anatomic and functional outcome, the practice is abandoned due to high complication rate [10]. Obliterative procedures have a higher success rate but should be recommended only for those who are elderly, medically compromised, and no longer sexually active [5]. Compared to vaginal hysterectomy with uterosacral ligament suspension, sacrospinous ligament suspension is found to have significantly less anatomic recurrence rate [11]. Sacrocolpopexy and SSLF are said to be efficient alternatives for the treatment of apical defect. Sacrocolpopexy has a better anatomic durability and sexual function but has higher rate complications making SSLF a better option [12]. In addition, the cost is significantly lower for SSLF [13]. In elderly and non-sexual active women, colpocleisis is better than vaginal hysterectomy having fast recovery, lower morbidity and higher success rate [14]. Doing hysterectomy during colpocliesis increases operative time with no advantage [15]. Though laparoscopic procedures are luxury for low resource limited countries, laparoscopic sacrocolpopexy is said to have a better recovery rate with lower blood loss than open abdominal sacrocolpopexy [16]. The modified abdominal high uterosacral colpo/hysteropexy, which is also being practiced in some areas of Ethiopia, is also a safe and effective treatment option for apical prolapse [17].

## Materials and methods

A cross-sectional study was conducted among Gynecologists in Ethiopia from January to June 2021. There were 450 Gynecologists registered in the Ethiopian Society of Obstetricians and Gynecologists during the time of data collection (ESOG office data, 2021) out of which 15 were Urogynecologists, 13 were urogynecology fellows and 20 Gynecologists who have received short term special training on management of pelvic organ prolapse at different times. All of the Gynecologists were approached via the ESOG group email address, telegram group and phone contact after notified by the president of the society. Those who were not accessible and those who have stopped practice during data collection were excluded from the study. Data was collected by an online Google form using structured questionnaires prepared in English. The tool contained the demographic characteristics of the participants, experience, patient encounter, methods of evaluation of prolapse patients, the type of the procedure they do for each compartment defect, and the reasons why they are not doing other procedures if any. Pretest was done on 20 Gynecology specialty residents in University of Gondar. The link of the Google form was sent via email and telegram. Study participants were communicated three times via notification emails, texts, telegram, and direct phone call. Those who were not willing to fill the form after three contacts were excluded. Those who had difficulty of filling the form due to internet problems were also excluded. Data were checked, coded and entered in to Epi info 7 and exported to SPSS version 22 software for further analysis. Frequencies were used to summarize descriptive statistics of the data. Tables were used for data presentation.

## Results

280 out of the 450 gynecologists practicing in Ethiopia have responded to the questionnaires resulting 62% response rate. About 10% were female gynecologists and 27% of the respondents practice in institutions with specialized center for care of urogynecologic cases. Thirty six (12.9%) have received short term special training on common surgical treatment options of prolapse.

### Surgical management practice of anterior compartment prolapses among Ethiopian gynecologists

Out of the 280 gynecologists 98.6% perform anterior colporrhaphy for the treatment of anterior vaginal wall prolapse. Abdominal and vaginal paravaginal repair is performed only by 3.2% and 0.7% of them respectively. Of those who are not performing paravaginal repair, 42.5% said they do not know the technique as their main reason not to perform it. Lack of appropriate material was the second reason mentioned by 19.6% of these professionals (Table 1).

**Table 1:** Surgical Management of Anterior vaginal wall prolapse among Ethiopian gynecologists

*Variables*	*Number*	*Percent (%)*
***Surgical management option performed by ObGyn for Anterior Vaginal Wall Prolapse (No 300, multiple responses)***		
Anterior colporrhaphy	276	98.6
Abdominal paravaginal repair	9	3.2
Vaginal paravaginal repair	2	0.7
Augmented repair with mesh	4	1.4
Augmented repair with graft	0	0
Other(*sacrospinus ligament fixation*)	9	3.2
***Reason for not performing Anterior colporrhaphy (n = 4) – Multiple responses***		
I didn’t know how to do it	1	25
Lack of the appropriate material	3	75
I feel the complication rate is high	2	50
I feel it is le effective than other alternatives	3	75
***Reason for not performing Paravaginal repair***		
I didn’t know how to do it	119	42.5
Lack of the appropriate material	55	19.6
I feel the complication rate is high	17	6
I feel it is less effective than other alternatives	13	4.6
Others (never encounter, patients are not available)	2	0.7

### Surgical management practice of apical compartment prolapse by Ethiopian gynecologists

Vaginal hysterectomy with McCall’s cul-do-plasty was performed by 51.8% of Ethiopian Gynecologists for the treatment of apical compartment defect. Vaginal hysterectomy with uterosacral ligament suspension was the second (49.6%) and sacrospinous ligament fixation and sacrocolpopexy were performed in 32.9% and 9.3% respectively. Of those who do not perform McCall’s cul-do-plasty 52.6% mentioned lack of skill as their reason not to do the procedure. Lack of skill was the commonest reason not to perform sacrospinous ligament fixation mentioned by 53.2% of the respondents and lack of appropriate material was the second reason (36.7%). Lack of skill is also reported by 66.5% of the responders who do not perform sacrocolpopexy and only 4 responders claim their reason to be high complication rate compared to vaginal procedures (Table 2).

**Table 2:** Surgical Management of Apical vaginal wall prolapse among Ethiopian gynecologists

*Variables*	*Number*	*Percent (%)*
***Surgical management option performed by ObGyn for Apical prolapse(central defect) (multiple responses)***		
Vaginal hysterectomy + McCall Culdoplasty	145	51.8
Vaginal hysterectomy with Uterosacral ligament suspension	139	49.6
Iliococcygeus suspension	3	1.1
Sacrospinous ligament fixation(SSLF)	92	32.9
Sacral colpopexy	26	9.3
Other(Colpocliesis)	25	8.9
***Reason for not performing McCall Culdoplasty (n = 135)***		
I didn’t know how to do it	71	52.6
Lack of the appropriate material	15	11.11
I feel complication rate is high	2	1.48
I feel it is le effective than other alternatives	22	16.3
***Reason for not performing Sacrospinous ligament fixation (n = 188)***		
I didn’t know how to do it	100	53.2
Lack of the appropriate material	69	36.7
I feel complication rate is high	14	7.4
I feel it is le effective than other alternatives	4	2.13
Other –*limited cases*	1	0.53
***Reason for not performing Sacral colpopexy (n = 254)***		
I didn’t know how to do it	169	66.5
Lack of the appropriate material	73	28.7
I feel complication rate is high	35	13.78
I feel it is le effective than other alternatives	9	3.54
Other – high complication	4	1.57

### Surgical management practice of posterior vaginal wall prolapse by Ethiopian gynecologists

Posterior colporrahphy was the commonest procedure performed for surgical treatment of posterior vaginal wall prolapse mentioned in 97.5% of the gynecologists followed by site-specific repair in 23.9% (Table 3). Perineorrhaphy was mentioned in only 4 of the respondents (1.4%).

**Table 3:** Surgical Management of Posterior vaginal wall prolapse among Ethiopian gynecologists

*Variables*	*Number*	*Percent (%)*
***Surgical management option performed by Gynecologists for Posterior Vaginal Wall prolapse (multiple responses, no = 349)***		
Posterior colporrhaphy	273	97.5
Defect directed repair	67	23.9
Augmented repair with mesh	5	1.8
Augmented repair with graft	0	0
Others (anterior rectal Plication, Posterior colporrhaphy with perineorrhaphy, sacrospinous ligament fixation)	4	1.4

## Discussion

### Anterior compartment defect repair

Although anterior colporrhaphy is still the commonest procedure performed for the treatment of anterior vaginal wall prolapse by other countries, and had immediate satisfactory outcome [18] there are alternative approaches with low recurrence and better anatomic correction specifically when the defect is paravaginal which could be worsened by mid line plication of the pubocervical fascia during colporrhaphy, Anterior colporrhaphy is also said to have 40% recurrence rate [19] So, vaginal approach to the correction of paravaginal defect cystocele is said to be highly effective, [20] but the practice by Ethiopian gynecologists is low. Ethiopian gynecologists perform anterior colporrhaphy commonly even for those who have paravaginal defect. Most of the gynecologists also do not try to identify the specific defects during pelvic organ prolapse evaluation because they do same procedure irrespective of the type of prolapse.

### Apical compartment defect repair

Vaginal hysterectomy during prolapse surgery cannot correct the defect and should only be done for other gynecologic reasons [21] Compared to vaginal hysterectomy with uterosacral ligament suspension, sacrospinous ligament suspension has significantly less anatomical recurrences [11]. But most of the Ethiopian gynecologists are still performing vaginal hysterectomy as a treatment for uterovaginal prolapse. Abdominal sacral colpopexy (ASC) is taken to be the optimal treatment for apical prolapse and has good durability and quality of life performance, [22] but compared to sacrospinous ligament suspension, it has high complication rate and is expensive [12]. The 32.9% reported rate of prforming sacrospinous ligament suspension looks a little exajurated compared to the number of urogynecologist fellows and those with special short term training on prolapse managemet. This coul be because, gynecologists ho graduate from institutions with urogynecology center are exposed to the procedure and may reort that they can erform the pprocedures. Some gynecologists without special training also peror sacrospinous ligament suspension only by looking at or assist while teir friends do the procedure. Sacrocolpopexy is performed by few Ethiopian gynecologists that could be because they don’t have training to perform this procedure and the number of trained gynecologists or urogynecologists in the country is low. Though McCall cul-do-plasty seems to be efficient in preventing vaginal vault prolapse in primary repair after hysterectomy with minimal morbidity, its efficacy on treating advanced prolapse is poor [23]. While doing McCall cul-do-plasty, the appropriate level of the uterosacral ligament plication should be at or above the ischial spine having similar success rates with sacrospinous ligament fixation (SSLF), [23] but the practice by our responders is to use the very distal end of the uterosacral ligament which is already weakened and stretched out beyond its normal position, so it is less effective as well as less durable. High uterosacral ligament suspension is also associated with ureteric injury. So, sacrospinous ligament fixation (SSLF) is a safe and effective alternative for treating apical vault prolapse via a vaginal route with a low recurrence rate and is more feasible compared to abdominal approaches [23]. SSLF can be done with or without hysterectomy and this will make it the best option for Ethiopian setup where there are significant number of women with prolapse but didn’t complete their family size. Though it is the best option, it is practiced only by 32.5% of our respondents. This number is actually higher compared to the number of urogynecologists, fellows and those who had special training, which tells us that there are gynecologists who perform sacrospinous ligament suspension without having special training, but learning from their colleagues who were trained. Those who have taken their gynecology specialty in institutions where there is urogynecology center may respond as they are also able to perform sacrospinous ligament suspension. The main reasons for not performing sacrospinous ligament suspension were reported to be lack of skill and appropriate materials.

### Posterior compartment defect repair

Defect-specific posterior colporrhaphy has an equal or superior outcome compared to traditional posterior colporrhaphy with respect to the anatomic success of the repair, functional outcome, and improvement in quality of life [24]. The practice of defect specific posterior repair by Ethiopian gynecologists is low. Even though most patients with advanced posterior wall prolapse have an associated perennial laxity and almost always require perineorrhaphy, Ethiopian gynecologists perform it rarely. The is no current evidence to recommend the routine use of any graft in posterior vaginal wall repair [25]. And only 4 respondents have mentioned the use of such graft in posterior repair, these respondents have probably performed the procedure long ago.

### Clinical implication

This study finding shows that most Ethiopian Gynecologists are still sticking to the traditional methods of surgical treatment of pelvic organ prolapse. Though there are few areas where contemporary procedures are performed, they are not readily available for the majority of patients. Lack of skill and appropriate materials were the reasons not to perform the contemporary procedures by the gynecologists.

## Conclusion and recommendation

Most gynecologists in Ethiopian continue to perform vaginal hysterectomy with cul-do-plasty and colporrhaphy procedures for treatment of pelvic organ prolapse. The alternative means of prolapse management are not practiced widely due primarily to lack of skill and appropriate materials to perform the procedures. Implementing short term training on alternative and contemporary surgical treatment options of pelvic organ prolapse with provision of suitable materials and increasing the number of urogynecologists in the country in the long run holds the potential to enhance the standard of care of women with the condition.

### Research implication

Further research should be done to identify areas where gynecologists who have skill gap are found and other factors associated for further policy intervention.

### Strength and limitation

The strength of this study is it has included participants from all levels of experience, different teaching, district and private institutions, places where urogynecology training is started. The limitation of this study is, despite contacting some of the gynecologists repeatedly with email, phone call and telegram, they were not able to respond for the questionnaires that resulted in a 62% response rate. We also expected that all the gynecologists accessed will give us the data so we didn’t stratify the quota among different levels of institutions.

### Electronic supplementary material

Below is the link to the electronic supplementary material.

Supplementary Material 1

## Data Availability

The datasets generated and/or analyzed during the current study are available in the supplementary file repository.

## List of abbreviations

ASC: Abdominal sacrocolpopexy
ESOG: Ethiopian Society of Obstetricians and Gynecologists
POP: Pelvic organ prolapse
SSLF: Sacrospinous ligament fixation
